# G allele at −924 A > G position of FoxP3 gene promoter as a risk factor for tuberculosis

**DOI:** 10.1186/s12879-017-2762-5

**Published:** 2017-10-11

**Authors:** Elham Beiranvand, Saeid Abediankenari, Soghra Khani, Hamideh Mahmoodzadeh Hosseini, Sirous Zeinali, Behnoush Beiranvand, Mehdi Goudarzi, Sima Sadat Seyedjavadi

**Affiliations:** 1 Immunogenic Research Center, Faculty of Medicine, Mazandaran University of Medical Sciences, P.O.Bax, Sari, 48175 Iran; 2 Biotechnology Department, Pasteur Institute of Iran, Tehran, Iran; 3 Department of Medical Mycology, Pasteur Institute of Iran, Tehran, Iran; 4 Applied Microbiology Research Center, Baqiyatallah University Medical of Sciences, Tehran, Iran; 5 General physician, Bam University of Medical Sciences, Kerman, Iran; 6 Department of Microbiology, School of Medicine, Shahid Beheshti University of Medical Sciences, Tehran, Iran; 7 Department of Medical Mycology, Pasteur institute of Iran, Tehran, Iran

**Keywords:** Forkhead box protein 3, Gene polymorphisms, Regulatory T cells, Tuberculosis

## Abstract

**Background:**

Forkhead box protein 3 (FoxP3) is an important factor for development and function of Regulatory T cells (Treg). Studies have found an association between common gene polymorphisms in *FoxP3* and some infectious diseases. The aim of this study was to evaluate possible associations between two Single nucleotide polymorphisms (SNPs) in the promoter of the *FoxP3* gene to susceptibility to tuberculosis (TB) and the alteration of *Foxp3* gene expression.

**Methods:**

The pattern distribution of genotype at two position, −3279 A > C (rs3761548) and −924 A > G (rs2232365) on the promoter of *FoxP3* gene was evaluated using polymerase chain reaction-single specific primer (PCR-SSP) method in 183 tuberculosis patients and 183 healthy control. In addition the quantity of *FoxP3* gene expression at mRNA level was identified by the real-time PCR.

**Results:**

The frequency of G allele at −924 A > G was significantly higher was higher in TB patients (59.5%) than control group (39.5%) (*P* ≤ 0.05). In addition, our data viewed approximately 5- folds more *FoxP3* gene expression in female patients with GG genotype in comparison to female healthy cases with the same genotype (*P* ≤ 0.001). There was no statistically significant differences between the distribution pattern of −3279 A > C polymorphism in patients and healthy individuals along with it effect on the *FoxP3* gene expression among both groups (*P* > 0.05).

**Conclusions:**

Our outcome suggests that the −924 A > G polymorphism leads to enhance *FoxP3* gene expression and susceptibility to tuberculosis in the sex dependent manner. This event may rise the count of Treg cells and modulate the immune response against tuberculosis.

## Background

TB is a chronic infectious disease that remains a major global health problem, responsible for ill health among millions of people each year [1]. In 2013, there were an estimated 9 million new cases of TB that 1.5 million of them died and an estimated 1.1 million (13%) of the 9 million people who developed TB in 2013, were HIV-positive [2].

*Mycobacterium tuberculosis* (*M. tuberculosis*), which breaks the physical barriers of respiratory tract and reach the lung, are immediately phagocytized by alveolar macrophages and dendritic cells and it is the first event in the host-pathogen relationship. The immune response to *M. tuberculosis* is complex. Studies have shown that a wide variety of cell types, cell surface molecules and cytokines are involved in the regulation of the immune response to tuberculosis [3]. Cell-mediated immunity (CMI) has an important role in the protective response against *M. tuberculosis* [4–6]. Recently, it was identified that a subset of CD4 T cells expressing the transcription factor Foxp3, called Treg cells, play a critical role in the regulation of the immune response by secretion of anti-inflammatory cytokines such as IL-10 and Transforming Growth Factors-β (TGF-β) that decreased CD4 T cells and memory T cells activity [7–9]. Some studies have shown that Treg cells expressing FoxP3 are expanded in blood and disease sites in TB patients [7, 10]. Also some studies have shown that Treg cells have a key role in expansion of TB by suppression of effector T-cells [11, 12]. FoxP3 belongs to the family of transcription factors that play a role in various cellular processes and is essential factor for development and function of Treg cells [13–15]. Previous studies have demonstrated that *FoxP3* gene polymorphisms associated with human diseases such as malaria, hepatitis B-related hepatocellular carcinoma, autoimmune diseases, IPEX syndrome, preeclampsia, abortion and cancer [16–23]. However, little information exists on the relationship between *FoxP3* gene polymorphisms and expression with the susceptibility to infectious disease especially tuberculosis. According to what was said, we hypothesized two SNP in promoter of *FoxP3* gene is related to an increase in *FoxP3* gene expression and result in susceptibility to tuberculosis in our target population [24].

## Methods

### Study population

The current study was cross-sectional, case-control study, carrying out in the Mazandaran Health Center in north Iran. Total 183 HIV-free TB patients (including 99 males and 84 females; mean age 46.8 ± 20.4 years). In addition, 183 control subjects (112 males and 71 females; mean age 44.1 ± 23.1) without any clinical features and family history of TB were recruited to study matching based on age, gender, and ethnicity with TB cases. All TB patients were diagnosed according to the World Health Organization (WHO) criteria: hilar adenopathy on chest X-ray, an infiltrate, histological evidence of TB, positive smear and culture [3]. All participants were evaluated for HIV by serological tests, and HIV cases were excluded from study. The local ethics committee approved the study, and informed consent was obtained from all participants.

### Sample collection, RNA and DNA extraction

Ten ml of heparinized Blood specimen was drawn from each patient and control case, separately. To isolate Peripheral mononuclear cells, blood samples were centrifuged using Ficoll–Hypaque gradient density and Their DNA was extracted utilizing DNA extraction kit (Roche, Germany) in accordance with the manufacturer’s instruction. The DNA samples were stored at -80̊C for further investigation. Furthermore, to obtain RNA from Peripheral mononuclear cells, RNA extraction kit (Fermentase, Italy) was utilized. Then, the quantity and quality of purified DNA and RNA were assessed by spectrophotometer (Picodrop, UK) and gel electrophoresis, respectively.

### Polymorphism detection

In order to find desired SNPs, HapMap database (http://www.hapmap.org) was used [25]. Two SNPs, rs3761548 and rs2232365 located in promoter regions of the *FoxP3* gene were selected and their genotypes were assessed by the PCR-SSP method. PCR primer sequences for genotyping are outlined in Table 1. Human epidermal growth factor receptor [26] gene was used as an internal control. Briefly, PCR reaction in volume of 25 μl, we used, 50 ng DNA template, 200 μM of each dNTP (mixture of dATP, dTTP, dCTP, dGTP), 0.2 μM of each primer, 1.5 mM of MgCl2, 10 mM of Tris hydrochloride (pH 8.3), and 1 U of Taq DNA polymerase (Fermentas, Italy). Cycling conditions included an initial denaturation step of 94 °C for 4 min followed by 35 cycles of denaturation (94 °C for 30s), annealing (61 °C for 30s), and extension (72 °C for 40s), the last cycle also included a final extension step at 72 °C for 5 min. PCR products were separated on 1.5% agarose gel and visualized with ethidium bromide staining under an ultraviolet illuminator. To assess the reproducibility of the analysis about 10% of the samples were genotyped two times for each polymorphism and the results were 100% identical to the ones of the first genotyping attempt.

**Table 1:** Primers used for SNPs of *FoxP3* gene

Position	Method	Primer Sequences	Allele Phenotypes
−3279 A > C (rs3761548)		Forward: CTGGCTCTCTCCCCAACTGA Forward: TGGCTCTCTCCCCAACTGC Common Reverse: ACAGAGCCCATCATCAGACTCTCTA	A: 334 bp C: 333 bp
−924 A > G (rs2232365)	PCR-SSP	Forward: CCCAGCTCAAGAGACCCCA Reverse: GGGCTAGTGAGGAGGCTATTGTAAC Forward: CCAGCTCAAGAGACCCCG Reverse: GCTATTGTAACAGTCCTGGCAAGTG	A: 442 bp G: 427 bp
FoxP3 gene	qRT- PCR	Forward: CAGCTGCCCACACTGCCCCTAG Reverse: CATTTGCCAGCAGTGGGTAG	
GAPDH gene		Forward: CCAGGTGGTCTCCTCTGACTTCAACA Reverse: AGGGTCTCTCTCTTCCTCTTGTGCTCT	

### FOX3 gene expression analysis

To identify the expression level of FOX3 gene, the quantitative real time PCR method was used based on the light cycler system (bio-rad, USA). The reagent quantity and thermal condition of qRT- PCR were as similar as our previous study [27]. Briefly, 1 μg of isolated RNA was applied for cDNA synthesis kit (Fermentas, Italy) containing oligo dT primers and M-MLV reverse transcriptase in accordance with instruction’s guidline. GAPDH was tested as the reference gene. The primers for both described genes were listed in Table 1. To amplify the FoxP3 gene, 0.5 μl of each cDNA was added to 20 μl master mixture solution including 2_x_ cyber green solution and 0.5 μM of described primers. The PCR program was set up as an initial denaturation step of 4 min at 95 °C, 35 cycles of 20 s at 94 °C, 61 °C at 30 s as annealing tempreature for each gene and 20 s at 72 °C. The efficiency of all run was between 95% to 99%. The relative expression of FoxP3 gene mRNA was evaluated according to standard curve obtained from the specific target and housekeeping gene. Finally, Pfaffl method was applied for data analysis.

### Statistical analysis

To study differences in the allele frequencies and genotype distribution between the groups. We used Fisher exact and x^2^ analysis tests. *P*-value of adjusted odds ratio calculated by logistic regression analysis with 95% confidence interval (CI). Accordance of genotype distribution with Hardy–Weinberg equilibrium was assessed by an exact test. Statistical power calculations were performed using Epi Info v. 6.02 (CDC Atlanta USA).

## Results

### Comparison of *FoxP3* genotypes (−924 A > G, −3279 A > C) in both case and control groups

**Fig. 1:**
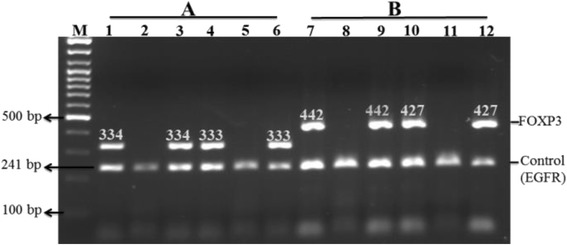
PCR-SSP analysis of −3279 A > C and −924 A > G SNP in the FoxP3 region. Three possible genotypes were defined by two distinct patterns of bands seen for each SNP on the gel (Every participant was presented by 2 lanes). A:-3279 A > C genotypes, Lanes 1, 2(AA), Lanes 3, 4 (AC) and Lanes 5, 6 (CC). B: -924 A > G genotypes, Lanes 7, 8 (AA), Lanes 9, 10 (AG) and Lanes 11, 12 (GG). Lane M indicates 100 bp molecular markers

Here, PCR-SSP method was applied to identify the two SNP, rs3761548 and rs2232365, at promoter region of *FoxP3* gene among 183 TB patients and 183 healthy subjects. Figure 1 depicts findings from gel electrophoresis of PCR products. Because the FoxP3 gene is localized in the small arm of the X-chromosome, data analyses were divided into female and male groups. Overall, in this case-control study, genotype distribution was in accordance with Hardy–Weinberg equilibrium. Furthermore, data revealed that allele G was significantly more prevalent in TB patients with compared to healthy group (OR = 0.43, 95% CI = 0.3 ± 0.61, *P* < 0.001). As outlined in Table 2, the prevalence of GG genotype was statistically higher in female patients than control group (OR = 0.35, 95% CI = 0.14 ± 0.85, *P* < 0.05). In addition, male patients had significantly higher G allele than control group (OR = 3.42, 95% CI = 2.17 ± 5.39, *P* < 0.01). However, there was no significant discrepant in the allele distribution at −3279 A > C between patients and control cases (*P* > 0.05).

**Table 2:** Genotypes and alleles frequencies of *FoxP3* gene SNPs in the cases and controls in relation to susceptibility to TB

Position	Gender (No)	Genotype/Allele	Controls (%)No	TB patient No (%)	Odd Ratioa (95% CI)	Pb
*FoxP3* −3279 A > C	Female	AA AC CC	38 (53.2) 20 (28.1) 13 (18.3)	43 (51.1) 15 (17.8) 26 (30.9)	1:00 (Reference)^c^ 0.56 (0.25 ± 1.2) 1.5 (0.67 ± 3.35)	0.1
	Male	A C	65 (58.03) 47 (41.9)	64 (64.6) 35 (35.3)	1:00 (Reference) 1.3 (0.75 ± 2.3)	0.3
	Total	A C	161 (63.3) 93 (36.6)	165 (61.7) 102 (38.2)	1:00 (Reference) 0.93 (0.65 ± 1.3)	0.7
*FoxP3* −924 A > G	Female	AA AG GG	34 (47.8) 14 (19.7) 23 (32.3)	19 (22.6) 22 (26.19) 43 (51.1)	1:00 (Reference) 0.29 (0.14 ± 0.63) 0.35 (0.14 ± 0.85)	0.005
	Male	A G	73 (65.1) 39 (34.8)	48 (48.4) 51 (51.5)	1:00 (Reference) 3.42 (2.17 ± 5.39)	0.01
	Total	A G	155 (61) 99 (39)	108 (40.4) 159 (59.5)	1:00 (Reference) 0.43(0.3 ± 0.61)	0.00

^a^Logistic regression analyses were used for calculating odds ratios with 95% confidence interval

^b^ *P* value was determined by x^2^ test (for genotype) or Fisher exact test (for alleles) from a 2 × 2 and 2 × 3 contingency table

^c^The first allele or genotype is considered as reference

### *FoxP3* gene expression according to *FoxP3* genotypes (−924 A > G, −3279 A > C)

**Fig. 2:**
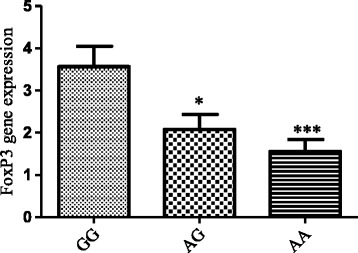
FoxP3 gene expression in female considering to genotype distribution pattern at −924 A > G position. There is statistically significant increase expression of FoxP3 gene in female group with GG genotype compared to other genotypes. The values shown are the means ±S.E.M. * p < 0.05 and *** *p* < 0.001 compared to GG genotype

Considering to discrepant frequency of alleles at −924 A > G in patients and control groups, we evaluated whether is there a relation between the genotype and the quantity of *FoxP3* gene expression? As our previous observation, *FoxP3* gene expression in tuberculosis patients was 2.8 fold higher than normal group (CI = 1.29 ± 2.37, *P* ≤ 0.001) [27]. Here, we observed statistically significant increase expression of *FoxP3* gene in female group with GG genotype compared to control group with the same genotype (P ≤ 0.001). Moreover, among female population, the *FoxP3* gene expression according to GG genotype was 2.28 folds and 1.79 fold more than the AA genotype (CI = 3.18 ± 0.84, *P* = 0.001) and AG genotypes (CI = 0.09 ± 2.87, *P* = 0.03), respectively (Fig. 2) However, there was higher *FoxP3* gene expression among patients groups compared with healthy subjects but among male population, the differences between the presence of A or G allele and case and control groups was not significant (*P* > 0.05(.In addition, there were no statistically significant differences between *FoxP3* gene expression and FoxP3–3279 A > C region in patients and control group (P > 0.05).

## Discussion

According to our knowledge, our work was the first finding of the assessment polymorphisms A to G in the promoter of *FoxP3* gene and its relation to susceptibility to tuberculosis. We observed that the presence of G allele in male and GG genotype in female patients with TB could be an effective marker increasing risk factor to emergence of TB. Several previous studies confirmed that Treg cells play critical roles in the development of TB and their number increases in peripheral blood and disease sites in patient [7–12].Since FoxP3 is the most specific molecular marker for naturally occurring Treg cells, therefore, many studies has been done on FoxP3 gene expression that some of them have shown increased *FoxP3* gene expression in patients with TB [27–29]. Our previous reports showed significant higher expression of *FoxP3* gene in TB patients. The combination of data from the *FoxP3* gene expression and SNP polymorphism provided the surprising finding. Here, our data viewed approximately 5- folds more *FoxP3* gene expression in female patients with GG genotype in comparison to female healthy cases with the same genotype. Hence, the harboring G allele particularly genotype of GG among female at the position of 924 on the promoter of *FoxP3* gene augments the risk of developing TB. So far, little information is available about *FoxP3* gene polymorphism in tuberculosis, but two studies reported that *FoxP3* gene polymorphism may be associated with hepatitis B-related and malaria [17, 19].

In summary, our outcome suggests that the −924 A > G polymorphism leads to enhance *FoxP3* gene expression and susceptibility to tuberculosis in the sex dependent manner. This event may rise the count of Treg cells and modulate the immune response against tuberculosis.

Nevertheless, these findings still remain unclear and Larger-scale studies involving multiple centers are needed to elucidate further the role of *FoxP3* gene polymorphism in tuberculosis. Also our study limitations include: a) In this study, only two SNPs of foxp3 gene were studied in patients and controls, but since there is still limited information about the relationship between foxp3 gene and tuberculosis, also with regard to the complexities observed in immune responses, it is recommended that other SNPs of this gene be investigated in patients who these studies are likely to help further clarify the interactions between the pathogen and the host. b) Some of the patients in Iran were infected with HIV but were excluded from our study. Therefore, it is suggested that in other studies, patients with TB and HIV co-infection should also be evaluated. c) In our study of the association between foxp3 gene polymorphisms in patients with active tuberculosis compared with control, it is suggested that this relationship be investigated in patients with latent tuberculosis infection.

## Abbreviations

CI: Confidence interval
CMI: Cell-mediated immunity
FoxP3: Forkhead box protein 3
*M. tuberculosis*: *Mycobacterium tuberculosis*
PCR-SSP: Polymerase chain reaction-single specific primer
SNPs: Single nucleotide polymorphisms
TB: Tuberculosis
TGF-β: Transforming Growth Factors-β
Treg: Regulatory T cells
WHO: World Health Organization
